# Thiol Carbazole Self‐Assembled Monolayers as Tunable Carrier Injecting Interlayers for Organic Transistors and Complementary Circuits

**DOI:** 10.1002/adma.202413157

**Published:** 2024-12-10

**Authors:** Mohamad Insan Nugraha, Yu‐Ying Yang, Zhongzhe Liu, George T. Harrison, Ryanda Enggar Anugrah Ardhi, Yuliar Firdaus, Qiao He, Linqu Luo, Mohamed Nejib Hedhili, Marco Thaler, Zhaoheng Ling, Matthias Zeilerbauer, Laerte L. Patera, Leonidas Tsetseris, Shadi Fatayer, Martin Heeney, Thomas D. Anthopoulos

**Affiliations:** ^1^ King Abdullah University of Science and Technology (KAUST) KAUST Solar Center (KSC) Thuwal 23955–6900 Saudi Arabia; ^2^ Research Center for Nanotechnology Systems National Research and Innovation Agency (BRIN) South Tangerang Banten 15314 Indonesia; ^3^ Collaboration Research Center for Advanced Energy Materials National Research and Innovation Agency – Institut Teknologi Bandung Jl Ganesha 10 Bandung 40132 Indonesia; ^4^ Research Center for Electronics National Research and Innovation Agency Bandung 40135 Indonesia; ^5^ Department of Chemistry and Centre for Processable Electronics Imperial College London White City Campus London W12 0BZ United Kingdom; ^6^ Core Labs King Abdullah University of Science and Technology (KAUST) Thuwal 23955–6900 Saudi Arabia; ^7^ Department of Physical Chemistry University of Innsbruck Innsbruck 6020 Austria; ^8^ Department of Physics School of Applied Mathematical and Physical Sciences National Technical University of Athens 9 Heroon Polytechniou Street, Zografou Campus Athens GR‐15780 Greece; ^9^ Applied Physics Program Physical Science and Engineering Division King Abdullah University of Science and Technology (KAUST) Thuwal 23955–6900 Saudi Arabia; ^10^ Henry Royce Institute and Photon Science Institute Department of Electrical and Electronic Engineering The University of Manchester Oxford Road Manchester M13 9PL United Kingdom

**Keywords:** organic thin‐film transistors, self‐assembled monolayers, thiol carbazoles, work function tuning

## Abstract

The significant contact resistance at the metal‐semiconductor interface is a well‐documented issue for organic thin‐film transistors (OTFTs) that hinders device and circuit performance. Here, this issue is tackled by developing three new thiol carbazole‐based self‐assembled monolayer (SAM) molecules, namely tBu‐2SCz, 2SCz, and Br‐2SCz, and utilizing them as carrier‐selective injection interlayers. The SAMs alter the work function of gold electrodes by more than 1 eV, making them suitable for use in hole and electron‐transporting OTFTs. Scanning tunneling microscopy analysis indicates that 2SCz and Br‐2SCz form highly ordered molecular rows, resulting in work function values of 4.86 and 5.48 eV, respectively. The latter value is higher than gold electrodes modified by the commonly used pentafluorobenzenethiol (≈5.33 eV), making Br‐2SCz promising for hole injection. Conversely, tBu‐2SCz appears disordered with a lower work function of 4.52 eV, making it more suitable for electron injection. These intriguing properties are leveraged to demonstrate hole‐ and electron‐transporting OTFTs with improved operating characteristics. All‐organic complementary inverters are finally demonstrated by integrating p‐ and n‐channel OTFTs, showcasing the potential of this simple yet powerful contact work function engineering approach. The present study highlights the versatility of thiol carbazole SAMs as carrier injecting interlayers for OTFTs and integrated circuits.

## Graphical abstract

Thiol‐based carbazole self‐assembled monolayers are synthesized and used to tune the work function of gold electrodes from 4.52 to 5.48 eV, making them suitable for application as hole or electron injecting contacts in organic thin‐film transistors. Using these thiol‐based monolayers, p/n‐channel organic transistors and complementary inverters are demonstrated.

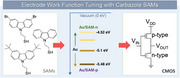

## 1 Introduction

Organic thin‐film transistors (OTFTs) are of great interest owing to their potential for application in flexible and low‐cost printed electronics.^[^ ^1^, ^2^, ^3^, ^4^, ^5^, ^6^, ^7^, ^8^, ^9^ ^]^ They serve as fundamental building blocks for diverse applications, which include wearable sensors,^[^ ^10^, ^11^, ^12^ ^]^ displays,^[^ ^13^, ^14^, ^15^ ^]^ flexible memories,^[^ ^16^, ^17^, ^18^ ^]^ radio frequency identification (RFID) tags,^[^ ^19^, ^20^ ^]^ and integrated circuits.^[^ ^21^, ^22^, ^23^ ^]^ However, charge injection at the metal electrode‐semiconductor interface is one of the main operational hurdles in OTFTs.^[^ ^24^ ^]^ Specifically, the injection barriers often present at the source and drain (S‐D) electrodes decrease injection efficiency, increase the contact resistance, and eventually reduce the performance of OTFTs by suppressing the effective potential across the transistor channel.^[^ ^25^, ^26^ ^]^ In OTFTs, the carrier injection is mostly determined by the mismatch between the work function of S‐D and the transport level of organic semiconductor(s).^[^ ^27^ ^]^ A large Schottky barrier between the work function of the electrodes and the transport level of the semiconductor increases the contact resistances and is one of the main factors limiting charge injection and hence transport across the device.^[^ ^24^ ^]^

In order to reduce the injection barriers at the contacts of OTFTs, different types of contact engineering methods and materials are typically used, including inserting a doping layer,^[^ ^28^ ^]^ a charge injection layer, or doping the bulk of the organic semiconductor.^[^ ^29^, ^30^, ^31^ ^]^ Surface modification of the S‐D contacts with polyethyleneimines,^[^ ^32^ ^]^ MoO_3_,^[^ ^33^ ^]^ barium salts,^[^ ^34^ ^]^ and self‐assembled monolayers (SAMs) has also been used to facilitate selective charge injection in OTFTs.^[^ ^35^, ^36^, ^37^ ^]^ Among the different interface modification strategies, surface functionalization with SAMs offers numerous attractive attributes.^[^ ^38^, ^39^, ^40^ ^]^ For example, their solution processability in various organic solvents makes them compatible with low‐temperature and cost‐effective processing techniques, such as immersion and spin‐coating methods. These techniques also allow the surface modification of electrode materials on temperature sensitive substrates without the need for high temperature or ultra‐high vacuum processing, highlighting the importance of SAMs for the realization of next‐generation low‐cost flexible optoelectronics.^[^ ^36^ ^]^ As a result, development of new types of SAMs with different functionalities is currently being pursued in order to improve the carrier injection and performance in p‐ and n‐type OTFTs.^[^ ^41^, ^42^, ^43^, ^44^ ^]^

In recent years, numerous studies on the development of new classes of SAMs have led to the discovery of phosphonic acid‐based carbazole SAMs such as 2PACz, Br‐2PACz, and MeO‐2PACz (their full chemical names are given in the supporting information).^[^ ^45^, ^46^, ^47^ ^]^ These SAMs have been extensively used to modify indium‐tin‐oxide (ITO) electrodes in optoelectronic devices. Phosphonic acid‐based carbazole SAMs can modify the work function of ITO, resulting in better charge extraction compared to other conventional hole‐transporting layers, thus enhancing the efficiency of organic and perovskite solar cells.^[^ ^45^, ^48^, ^49^, ^50^, ^51^, ^52^ ^]^ Despite their potential in solar cells, the use of carbazole SAMs in OTFTs has received limited attention. This is mainly because the majority of OTFTs utilize Au source‐drain electrodes, which are known to interact more favorably with thiol (SH) functional groups rather than phosphonic acid ones.

**Figure 1:**
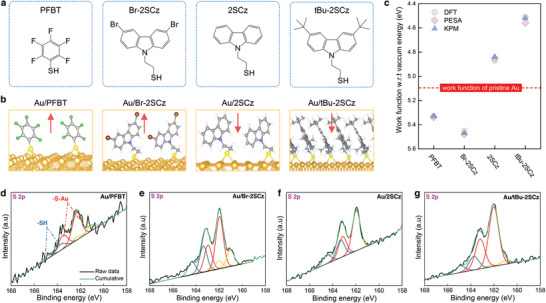
a) Molecular structures of PFBT, Br‐2SCz, 2SCz, and tBu‐2SCz SAMs, b) The attachment of thiol SAMs on Au films as predicted by Density Functional Theory (DFT) with the arrows indicate the direction of dipole moment induced by the SAMs, c) Work function of PFBT, Br‐2SCz, 2SCz, and tBu‐2SCz‐functionalized Au films obtained from DFT calculations, photoelectron spectroscopy in air (PESA), and Kelvin probe measurements (KPM), and X‐ray photoelectron spectroscopy (XPS) spectra of S 2p in d) PFBT‐, e) Br‐2SCz‐, f) 2SCz‐, and g) tBu‐2SCz‐treated Au films. Red and yellow plots correspond to the thiol‐Au (S─Au) bonds, while blue plots correspond to the physisorbed thiol molecules. Black and light green plots correspond to the raw and cumulative deconvoluted XPS data. The S─Au bonds are displayed by the red and orange plots, while the S─H bonds are shown by the blue plots.

Here, we develop carbazole‐based SAMs functionalized with a thiol anchoring group to modify the work function of Au electrodes. Materials synthesized include the unsubstituted thiol carbazole (2SCz), and its two derivatives, namely Br‐2SCz and tBu‐2SCz (Figure 1a) (full chemical names in SI). This molecular design enables us to adjust the work function of the Au electrodes within the range of 4.51–5.48 eV. All SAMs are found to bind to Au by anchoring of the thiol end‐groups, causing the observed work function changes. Surface functionalization of Au electrodes with Br‐2SCz results in the highest hole mobility in p‐type OTFTs, reaching up to 5.46 cm^2^V^−1 ^s^−1^, a value superior to that achieved with the widely used pentafluorobenzenethiol (PFBT) SAM. Surface functionalization of Au electrodes with tBu‐2SCz, on the other hand, yielded n‐type OTFTs with significantly improved electron mobility. Finally, we developed all‐organic complementary inverters with wide noise margins and high voltage gain by combining the p‐ and n‐type OTFTs featuring the thiol‐carbazole‐based SAMs‐treated Au electrodes. This work highlights the potential of thiol carbazole SAMs to effectively modify the carrier injection characteristics of Au electrodes, making them attractive for application in emerging optoelectronics.

## 2 Results and Discussion

To date, PFBT SAMs have been widely used to functionalize Au films, with a reported work function changes from 5.09‐5.8 eV.^[^ ^26^, ^39^, ^53^, ^54^, ^55^, ^56^ ^]^ Here, the work function of Au‐PFBT electrodes consistently yield values ≈ 5.30 eV; an experimental finding corroborated by Density Functional Theory (DFT) calculations that will be discussed later. To further enhance the hole injection properties in p‐type OTFTs and fine‐tune the work function of Au electrodes over a wider range, we designed several new thiol SAM molecules, namely Br‐2SCz, 2SCz, and tBu‐2SCz (**Figure** **1**a). The detailed methods for the synthesis of thiol SAM molecules are provided in the *Supplementary Information*. The structures were confirmed by a combination of ^1^H and ^13^C nuclear magnetic resonance (NMR) spectroscopy and high‐resolution mass spectroscopy (HRMS) as shown in Figures S1‐S9 (Supporting Information). Figure 1b illustrates the attachment of thiol SAM molecules to the Au surface, as predicted by DFT calculations. The individual SAM molecule is effectively attached to the Au surface by anchoring the sulphur (S) end group of the SAM molecule with Au atoms.

We performed DFT calculations to investigate the electronic structures of Au functionalized with different thiol SAMs. In particular, we aimed to identify atomic‐scale SAM configurations whose corresponding calculated work functions are in close agreement with pertinent experimental data. The calculated electrostatic surface potentials of the Au films functionalized with different thiol SAMs are shown in Figure S10 (Supporting Information). Upon surface functionalization with PFBT SAMs, the work function of Au is predicted to deepen from −5.1 to −5.33 eV, in agreement with numerous experimental reports.^[^ ^26^, ^37^ ^]^ Functionalization with the carbazole SAMs was also predicted to result in significant changes in work function, with a strong dependency on the nature of the functional group on the carbazole. Thus Br‐2SCz was predicted to result in a further deepening of the work function to 5.44 eV, potentially improving the hole injection characteristics in p‐type OTFTs compared to those with PFBT SAMs. In contrast, for 2SCz and tBu‐2SCz SAMs, we found that the predicted work function of Au was shallower than that of pristine Au, i.e., 4.86 and 4.51 eV, respectively. The changes of the work function in Au films upon functionalization with different thiol SAMs is attributed to the formation of dipole moments on the Au surface. The direction of the dipole moments induced by the SAMs is illustrated by the arrows in Figure 1b, with their values are given in Table S1 (Supporting Information). PFBT and Br‐2SCz SAMs induced upward dipole moments, while 2SCz and tBu‐2SCz resulted in downward dipole moments on Au films. These results are consistent with the calculated values of the Au work functions and with the electronegativity or electropositivity of the adsorbed molecules on the Au films.

To experimentally verify the work function values obtained from DFT calculations for the various Au/SAM electrodes, we performed photoelectron spectroscopy in air (PESA) and Kelvin probe measurements (KPM) (Figures S11 and S12; Table S1, Supporting Information). The obtained values along the DFT calculations are summarized in Figure 1c. All three methods yielded consistent values, confirming the validity of the measured work functions. Specifically, the experimental measurements revealed that the work function of Au can be tuned from 5.48 eV (Br‐2SCz), 5.34 eV (PFBT), 4.85 eV (2SCz), to 4.52 eV (tBu‐2SCz).

The adsorption of thiol SAMs on Au was further studied using contact angle measurements. As summarized in Table S2 and Figure S13 (Supporting Information), pristine Au films exhibit a water contact angle of ≈66.1° and surface energy of ≈43.1 mN m^−1^. Upon Au surface functionalization with thiol SAMs, the contact angles increase to ≈80.5° (PFBT), ≈74.9° (Br‐2SCz), ≈76.5° (2SCz), and ≈91.1° (tBu‐2SCz), with corresponding surface energies of 32.7, 37.6, 35.5, and 29.6 mN m^−1^ respectively. The large surface energy changes observed indicate successful immobilization of the SAMs.

Further confirmation of the presence of SAMs on the surface of Au was obtained by ellipsometry measurements (samples: Si/SiO_2_(48 nm)/Al(5 nm)/Au(79 nm)/SAMs). The optical constants of the SAMs, inferred from these measurements, are presented in Figure S14 (Supporting Information). PFBT exhibited a main peak in the extinction coefficient plot at 338 nm and an absorption onset at 2.98 eV. Due to the longer conjugation length of the carbazole‐based SAMs, the absorption onsets are shifted to lower energies (≈2.48 eV). The thickness of PFBT monolayer extracted from ellipsometry analysis is 0.66 nm and close to that predicted from the DFT calculation (0.74 nm). As expected, the thiol carbazole SAM appears thicker than PFBT (Table S3, Supporting Information). Interestingly, the thicknesses of the carbazole SAMs are roughly twice those estimated from DFT calculations (Table S3, Supporting Information). This suggests the co‐existence of physisorbed thiol carbazole molecules and a monolayer of bound thiol carbazole molecules, as supported by X‐ray photoelectron spectroscopy (XPS) measurements (vide infra). We also examined the surface morphology of the thiol SAMs‐treated Au films using scanning electron microscopy (SEM) measurements. As shown in Figures S15 and S16 (Supporting Information), we did not observe any obvious changes in the morphology of the Au films after surface functionalization with different thiol SAMs. This indicates that the deposited SAMs form well‐distributed layers on Au, making them good candidates for electronic applications.

We also performed XPS measurements to identify the chemical state of the thiol SAM molecules and confirm their presence on the Au surfaces. Upon surface functionalization with PFBT, a strong peak was observed in the XPS spectra of the F 1s core level of the PFBT‐treated Au films at ≈687.7 eV (Figure S17a, Supporting Information). With the thiol carbazole SAMs, first we observed two doublet peaks in the XPS spectra of the Br 3d core level at ≈69.7 and ≈70.7 eV corresponding to Br 3d_5/2_ and Br 3d_3/2_, respectively, in the Br‐2SCz SAMs‐treated Au films (Figure S17b, Supporting Information). The Br‐2SCz SAMs‐functionalized Au films also exhibited a strong peak in the XPS spectra of the N 1s core level at ≈399.5 eV (Figure S17c, Supporting Information). These results are in line with the molecular structures of PFBT and Br‐2SCz SAMs illustrated in Figure 1a, which possess F atoms in the former and Br and N atoms in the latter. Meanwhile, the presence of 2SCz and tBu‐2SCz SAMs on Au was revealed by the observed peak in the XPS spectra of the N 1s core level at ≈399.5 eV (Figures S17d,e, Supporting Information).

To investigate the interaction of the SAM molecules with the Au surfaces, we analyzed the high‐resolution XPS spectra of the S 2p chemical states (Figure 1d–g). The core level spectra were fitted using three doublets S 2p_3/2_ – S 2p_1/2_ with a fixed area ratio equal to 2:1 and doublet separation of 1.18 eV. The binding energy of the S 2p_3/2_ component at ≈162.0 eV corresponds to S–Au bonding through the thiol sulfur.^[^ ^58^, ^59^ ^]^ The S 2p_3/2_ component at ≈163.3 eV is attributed to the ─SH bond from physisorbed thiol SAM molecules on the Au surface.^[^ ^58^, ^59^ ^]^ The S 2p_3/2_ component at ≈161.0 eV is associated with atomically adsorbed sulfur, which could result from SAM degradation or impurities present in thiol SAMs.^[^ ^59^ ^]^ At this point, we can infer that PFBT and all thiol carbazole SAMs‐treated Au films possess both S─Au bonds and physisorbed molecules. From the XPS measurements, we also observe that the S─Au covalent bonding is the dominant contribution to the S 2p core level spectra, confirming that a chemical thiol‐gold bond is formed by breaking S─H bonds near the interface (confirmed also by the DFT calculations). These S─Au covalent bonds modify the electronic structures of the Au surface and change its work function as verified by the PESA and KPM measurements.

**Figure 2:**
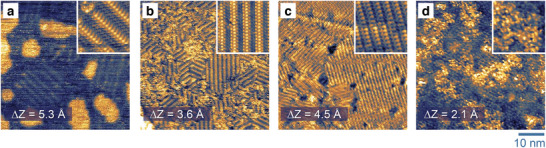
Scanning tunneling microscopy (STM) constant‐current images of: a) PFBT, b) Br‐2SCz, c) 2SCz‐, and d) tBu‐2SCz‐functionalized Au (111) single crystal surface. The size of inset images is 7 × 7 nm^2^. Imaging parameters: a. *V* = 250 mV, *I* = 50 pA (inset *V* = 200 mV, *I* = 30 pA), b. *V* = 200 mV, *I* = 3 pA (inset 200 mV, *I* = 5 pA), c. *V* = 150 mV, *I* = 20 pA (inset *V* = 150 mV, *I* = 30 pA), d. *V* = 1000 mV, *I* = 20 pA. Images have been acquired at a temperature of 8.5 K a–c) and 300 K d). Scale bar (for all images): 10 nm.

Direct evidence of the presence of thiol SAMs on Au were obtained using scanning tunneling microscopy (STM) measurements following ex situ deposition of the thiol SAMs via dip coating. **Figure** **2** displays ultra‐high vacuum STM images of different thiol SAMs on a model Au (111). Similar to the literature,^[^ ^60^ ^]^ PFBT yielded long‐range and well‐ordered monolayers on Au films, as highlighted by their row formation (Figure 2a). There are brighter regions in the STM image whose apparent height is ≈0.264 nm, similar to the Au (111) monoatomic height, which constitutes gold adatom islands (see Figure S18, Supporting Information).^[^ ^60^ ^]^ From the images in Figure 2b,c, we also observed that Br‐2SCz and 2SCz monolayers display ordered phases composed of parallel rows. The measured spacing between rows is 1.7 ± 0.5 nm for Br‐2SCz and 1.3 ± 0.4 nm for 2SCz, as determined by fast Fourier transformation (FFT) analysis (see Figure S19, Supporting Information for more details). Within the rows, there are repeating units with a spacing of 0.4 ± 0.1 nm for Br‐2SCz and 0.5 ± 0.1 nm for 2SCz. The repeating units are observed within the row, and are attributed to the individual molecules. Further details on the superlattice formed by the Br‐2SCz and 2SCz on Au (111) are provided in the Supporting Information. According to DFT calculations, all thiol SAMs are upstanding, with each carbazole being perpendicular to the Au (111) surface in agreement with the ellipsometry and STM analysis. Based on the STM images (Figure 2d), the tBu‐2SCz did not order on Au (111), hinting at the added steric hindrance from the tert‐butyl substituents as a cause for the disorder. The packing motif may also be impacted by the different deposition conditions used for tBu‐2SCz as the material was processed from the non‐polar toluene.

**Figure 3:**
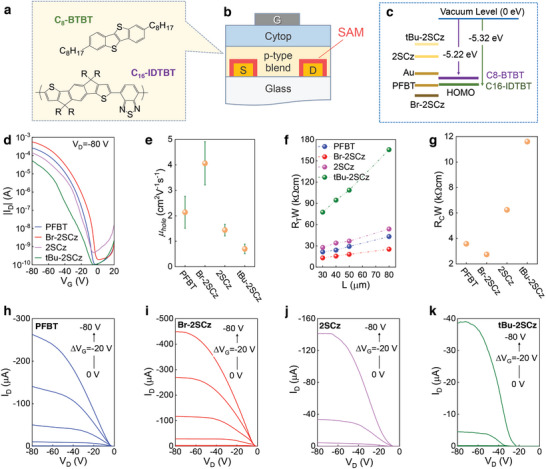
a) Molecular structures of C16‐IDTBT and C8‐BTBT employed as p‐type active layers in OTFTs, b) schematic of OTFTs with different surface functionalized Au S/D electrodes, c) energy band diagram of C16‐IDTBT, C8‐BTBT, and different surface functionalized Au films. d) Representative set of transfer characteristics, e) mean hole mobility value with the standard deviation, f) total resistance versus channel length, g) contact resistance, and h–k) output characteristics of C16‐IDTBT:C8‐BTBT blend OTFTs with different surface functionalized Au S/D electrodes. The channel length/width dimensions for the OTFTs shown in Figure 3d,h–k were 30/1000 µm.

Next we studied the impact of source‐drain (S/D) functionalization with the different SAMs on the electrical characteristics of the OTFTs. The hole‐transporting C16‐IDTBT:C8‐BTBT organic blend (**Figure** **3**a) was chosen to construct the p‐channel transistors due to its high carrier mobility.^[^ ^57^ ^]^ The top‐gate bottom‐contact transistor architecture was chosen with Cytop as the gate dielectric due to its solvent orthogonality and hydroxyl‐free nature, which enables the realization of trap‐free channel interfaces that are difficult to achieve using conventional dielectrics such as SiO_2_ (Figure 3b). Figure 3c shows the energy level diagram illustrating the highest occupied molecular orbital (HOMO) of the organic semiconductors and the work functions of different thiol SAMs‐functionalized Au electrodes. The C16‐IDTBT and C8‐BTBT organic semiconductors have HOMO levels of −5.22 and −5.32 eV, respectively, as measured by PESA (Figure S20, Supporting Information). On the other hand, Au/PFBT electrodes exhibit a work function of −5.34 eV, making them a good choice for hole injection into the HOMO levels of the blend semiconductor (i.e. C16‐IDTBT:C8‐BTBT). Functionalization of Au with Br‐2SCz yields a larger work function of 5.48 eV, which is deeper than that of Au/PFBT and the HOMO levels of C8‐BTBT and C16‐IDTBT, making the particular electrode very promising for hole‐injection. On the other hand, the work functions of Au/2SCz and Au/tBu‐2SCz electrodes were shallower than the HOMO energy of the semiconductor blend, resulting in an increase in the injection barrier for holes.

The transfer characteristics of the C16‐IDTBT:C8‐BTBT blend OTFTs employing the different Au/SAM electrodes are depicted in Figure 3d. The C16‐IDTBT:C8‐BTBT blend OTFTs exhibited p‐type transport characteristics with on/off ratios of >10^5^. Upon functionalization of Au with PFBT, the channel on‐current increased compared to OTFTs featuring the untreated (bare) Au electrodes (Figure S21, Supporting Information). Further improvements in the transfer characteristics of the C16‐IDTBT:C8‐BTBT blend OTFTs were obtained upon functionalization of the Au electrodes with Br‐2SCz. The ensuing p‐type OTFTs exhibited the highest on‐current (>10^6^) among devices employing Au electrodes treated with the different SAMs. The significant improvement in the electrical characteristics of the devices with Au/Br‐2SCz electrodes indicates efficient hole injection to the semiconducting channel. Conversely, transistors based on Au/2SCz show reduced channel current. A further reduction in the device current was observed when Au/tBu‐2SCz electrodes were used. The latter OTFTs exhibit non‐ideal transfer characteristics manifested as non‐linear current‐voltage characteristics at low source‐drain bias, indicative of an injection‐limited process. Compared to transistors with PFBT and Br‐2SCz, devices featuring the 2SCz and tBu‐2SCz SAMs exhibited larger hysteresis (Figure S22, Supporting Information), with the Au/tBu‐2SCz OTFTs exhibiting the largest.

We further analyzed the hole mobility (*µ_h_*) of the C16‐IDTBT:C8‐BTBT blend OTFTs employing Au electrodes functionalized with the various thiol SAMs (Figure 3e). The *µ_h_* was calculated in the saturation regime using:

(1) $$\mu_{h}=\frac{2L}{W}\;{\left(\frac{d{I_{D}}^{1/2}}{dV_{G}}\right)}^{2}$$

where *L*, *W*, *I_D_*, and *V_G_* denote the channel length, channel width, source‐drain current, and gate voltage, respectively. Figure S23 (Supporting Information) shows the I_D_^1/2^ versus V_G_ characteristics for devices treated with different SAMs from which the *µ_h_* was calculated using Equation (1). The linearity of the plots supports the validity of the gradual channel approximation used for the carrier mobility analysis. OTFTs featuring Au/PFBT electrodes exhibited an average hole mobility of 2.14 cm^2^V^−1^s^−1^, in agreement with previous reports.^[^ ^57^, ^61^ ^]^ Devices based on Au/Br‐2SCz electrodes showed increased average/maximum hole mobility values of 4.1/5.46 cm^2^V^−1^s^−1^, respectively. For transistors with Au/2SCz electrodes the hole mobility decreased to 1.45 cm^2^V^−1^s^−1^. A further reduction in the average hole mobility to 0.7 cm^2^V^−1^s^−1^ was observed in devices with Au/tBu‐2SCz electrodes. The observed trend in *µ_h_* for the various thiol SAMs is in line with the energy level offset present between the work function of Au and the HOMO levels of the blend semiconductor (Figure 3c). Functionalization with Br‐2SCz shifts the work function of Au to 5.48 eV, which is deeper than the HOMO of both C8‐BTBT and C16‐IDTBT. The favorable interface energetics improve the hole injection to the semiconducting channel, ultimately leading to the higher channel current and *µ_h_* observed. On the other hand, applying tBu‐2SCz to Au shifts its work function to 4.52 eV, which is significantly shallower than the HOMO levels of C8‐BTBT and C16‐IDTBT. This considerable energy offset gives rise to a large Schottky barrier for holes and to a lower *µ_h_* in the OTFTs based on Au/tBu‐2SCz.

To elucidate the role of the various SAMs on the transistors’ carrier injection characteristics, we analyzed the contact resistance using the transmission line method (TLM). The method allows for the extraction of the total resistance (*R_T_*), which is composed of the channel resistance (R_ch_) plus the contact resistance (R_c_), and is given as *R_T_* = *R_ch_* + 2*R_c_*. Figure 3f displays the total resistance normalized to the channel width (*R_T_* ∙*W*) versus the channel length for OTFTs featuring the different thiol SAMs. Devices with Au/Br‐2SCz electrodes exhibited the lowest *R_T_* ∙*W*; lower than Au/PFBT‐based OTFTs. On the other hand, transistors with Au/tBu‐2SCz electrodes exhibited the highest *R_T_* ∙*W* values, an effect attributed to the electrode's low work function and the ensuing injection barrier for holes. The *R_C_* was also calculated by extrapolating the linear part of the *R_T_* ∙*W vs. L* plot to the origin. Figure 3g displays the *R_C_* for C16‐IDTBT:C8‐BTBT transistors based on the different thiol SAMs. Compared to the bare Au devices (5.0 kΩ∙cm), OTFTs featuring Au/PFBT show reduced contact resistance of 3.58 kΩ∙cm. The *R_C_* decreases further to 2.74 kΩ∙cm when Au/Br‐2SCz electrodes are employed ultimately leading to the highest *µ_h_* achieved. On the other hand, applying 2SCz and tBu‐2SCz to Au increases the *R_C_* to 6.24 and 11.6 kΩ∙cm, respectively. The higher R_C_ values are ascribed to the shallower work function of these electrodes and the larger injection barrier for holes.

The differences in the charge‐transporting properties of the studied OTFTs are also evidenced in their output characteristics (Figure 3h–k). For example, compared to devices with Au/PFBT (Figure 3h), transistors based on Au/Br‐2SCz exhibit higher currents which is indicative of higher carrier mobility (Figure 3i). The latter OTFTs also displayed improved Ohmic contact behavior than those made with Au/PFBT. In contrast, for Au/2SCz based devices, we observed s‐shaped (non‐linear) output characteristics at low drain voltages (Figure 3j). The latter feature becomes more apparent in OTFTs with Au/tBu‐2SCz and is accompanied by a significant channel current decrease (Figure 3k). The characteristic s‐shaped curves and reduced currents are attributed to the presence of injection barriers due to energetic offsets discussed earlier. Meanwhile, using Au/Br‐2SCz electrodes yields nearly linear output curves and higher channel current indicative of Ohmic‐like hole‐injecting characteristics.

**Figure 4:**
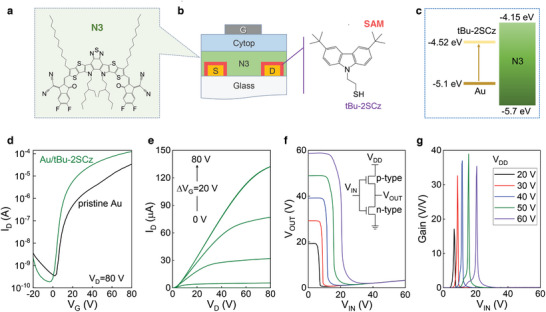
a) Molecular structure of N3 employed as active layers in OTFTs, b) Schematic of N3 OTFTs with tBu‐2SCz functionalized Au S/D electrodes, c) Energy band diagram of N3 and tBu‐2SCz SAMs‐functionalized Au films. d) Transfer characteristics of N3 TFTs with pristine and tBu‐2SCz SAMs‐functionalized Au S/D electrodes. e) Output characteristics of N3 TFTs with tBu‐2SCz SAMs‐functionalized Au S/D electrodes. f) The output and g) gain characteristics of complementary inverter employing Br‐2SCz‐Au and tBu‐2SCz SAMs‐treated Au in C16‐IDTBT:C8‐BTBT and N3 OTFTs, respectively. The channel length and width for the devices with electrical characteristics shown in Figure 4d–g are 30 and 1000 µm, respectively.

Although Au/tBu‐2SCz electrodes appear unsuitable for p‐type OTFTs, their low work function makes them promising for use as electron‐injecting contacts in n‐type OTFTs. To test this hypothesis, we selected the small‐molecule N3 (**Figure** **4**a) as the n‐type organic semiconductor to fabricate the OTFTs.^[^ ^31^ ^]^ A schematic structure of the OTFTs featuring Au/tBu‐2SCz electrodes is shown in Figure 4b. The LUMO level of N3 was measured via PESA (Figure S20, Supporting Information) to be ≈−4.15 eV, while its optical band gap was estimated to be ≈ 1.55 eV. These measurements suggest the presence of a substantial injection barrier for electrons between the bare Au and the LUMO of N3 (Figure 4c). Functionalizing the Au electrodes with tBu‐2SCz shifts the electrode work function to ≈4.52 eV reducing the potential barrier for electrons from 0.9 to 0.37 eV.

The transfer characteristics of N3‐based OTFTs employing bare Au and Au/tBu‐2SCz electrodes are presented in Figure 4d. The devices displayed clear electron transport behavior with a current modulation of over 10^5^. Transistors based on bare Au electrodes showed non‐ideal transfer characteristics indicative of the presence of a large injection barrier for electrons originating from the deeper work function of Au leading to non‐ideal characteristics. As a result, it was difficult to reliably extract the electron mobility (*µ_e_*) for these devices. As shown in Figure S24 (Supporting Information), the electron mobility in the N3 OTFTs with pristine Au electrodes can vary from 0.05 to 0.22 cm^2^V^−1^ s^−1^ depending on the interval of gate voltage used for the fitting. Upon functionalization of the Au electrodes with tBu‐2SCz, we observed a significant increase in the channel current, with an on/off ratio of >10^6^. In OTFTs with tBu‐2SCz electrodes, the *µ_e_* increases significantly to 0.51 cm^2^V^−1^s^−1^. We attribute this to the lower *R_C_* of the devices, which is found to decrease from 168.3 to 64.2 kΩ∙cm upon Au functionalization with tBu‐2SCz (Figure S25, Supporting Information). Overall, the increased channel current, the higher *µ_e_*, and the lower *R_C_* indicate a drastically improved electron injection for the Au/tBu‐2SCz based devices compared to transistors featuring bare Au electrodes (Figures 4e and S24, Supporting Information).

The promising attributes of the new thiol carbazole SAMs were explored to develop a complementary inverter consisting of p‐type (C16‐IDTBT:C8‐BTBT) and n‐type (N3) OTFTs. These proof‐of‐concept circuits were created by connecting best‐performing OTFTs located on different substrates to form the equivalent circuit shown in the inset of Figure 4f. The p‐type OTFT featured Au/Br‐2SCz electrodes, while the n‐type device used Au/tBu‐2SCz electrodes. The voltage transfer characteristics of a representative complementary inverter measured at different *V_DD_* biases are shown in Figure 4f. The circuits exhibit sharp switching at varying trip voltages depending on *V_DD_*, indicating successful input signal inversion. The voltage gain, given as *ΔV_OUT_/ΔV_IN_*, versus V_IN_ at different *V_DD_*, is plotted in Figure 4g. The circuit's signal gain gradually increased with increasing V_DD_ to 50 V, reaching a maximum value of 38.9 V/V, which compares favorably with previous reports on C16‐IDTBT‐based complementary inverter.^[^ ^62^, ^63^, ^64^ ^]^ Further advances are anticipated using thinner and/or higher‐k gate dielectrics combined with improved n‐channel materials/OTFTs. Monolithically integrated complementary circuits are also possible by exploiting recent advances in processing schemes. For example, p‐ and n‐channel transistors can be manufactured on different levels on top of each other sequentially. The p‐ and n‐channel transistors located in different stacks can then be interconnected vertically using vertical interconnects to form complementary or unipolar circuits.^[^ ^66^, ^67^ ^]^ The latter approach reduces the need for high resolution patterning of the semiconductor while enabling SAM functionalization on the same metal electrodes without added complexity.

## 3 Conclusion

We have synthesized and used thiol carbazole‐based SAMs as carrier selective injection interlayers for Au electrodes in OTFTs. We found that Br‐2SCz, 2SCz, and tBu‐2SCz can modify the work function of Au electrodes over a wide energy range and help enhance the hole and electron injection characteristics of OTFTs based on different organic semiconductors. By combining DFT calculations, STM, spectroscopic measurements, and charge‐transport characterization, we elucidated the impact of the different thiol SAMs on the electronic properties of Au electrodes at the nanoscale and their influence on the operating characteristics of OTFTs. Incorporating Au/Br‐2SCz electrodes, in particular, yielded hole‐transporting OTFTs with improved hole mobility. Conversely, Au/tBu‐2SCz electrodes enhanced the electron injection from Au, yielding n‐channel OTFTs with well‐behaved operation. The complementary properties of these Au work function modifying SAMs were explored to demonstrate fully functional complementary inverters with promising operating characteristics. Our findings highlight thiol carbazole SAMs as a promising family of work function modifying agents for OTFTs and potentially other organic devices, including light‐emitting diodes, light‐emitting transistors, electrochemical transistors, and photodetectors.

## 4 Experimental Section

### Functionalization of Au Films with Thiol SAMs

The glass substrates were cleaned by sequential ultrasonication in dilute Extran 300 detergent solution and deionized water for 30 min each. The substrates were then cleaned with acetone and isopropyl alcohol by ultrasonication for 10 min each. Au (40 nm) with 5 nm Al as the adhesion layer was evaporated at 2 × 10^−6^ mbar. Before thiol SAM functionalization, Au‐coated glass substrates were subjected to UV‐ozone treatment for 15 min. For the PFBT SAM treatment, 10 µL PFBT was mixed with 10 mL isopropyl alcohol. The Au‐coated glass substrates were then immersed in PFBT solution for 30 min at room temperature, rinsed with isopropyl alcohol, and dried using an N_2_ gun. For Br‐2SCz and SCz SAM treatment, the solution of Br‐2SCz and 2SCz SAMs (0.3 mg mL^−1^) was prepared by dissolving the Br‐2SCz or 2SCz powder in absolute ethanol. For the tBu‐2SCz SAM treatment, the tBu‐2SCz powder was dissolved in anhydrous toluene (0.3 mg mL^−1^). All solutions were subsequently ultrasonicated at room temperature for 30 min to ensure complete dissolution. The Au‐coated glass substrates were then immersed in Br‐2SCz, 2SCz, or tBu‐2SCz SAM solutions for 5 min at room temperature. The films were then subjected to thermal annealing at 120 °C for 5 min inside an N_2_‐filled glove box.

### Fabrication of Organic Thin‐Film Transistors

N3 was purchased from 1‐Material Inc. C8‐BTBT was purchased from Luminescence Technology Corp. C16‐IDTBT was synthesized by the reported procedure,^[^ ^65^ ^]^ with an M_n_ of 65 kgmol^−1^ and a dispersity of 2.42 as measured by GPC in chlorobenzene at 80 °C against polystyrene standards. Glass substrates were cleaned using the method described above. As source‐drain electrodes, 35 nm of Au with 5 nm of Al as the adhesion layer was thermally evaporated at 2 × 10^−6^ mbar. The substrates with patterned Au/Al source‐drain contacts were subjected to a UV‐ozone treatment for 15 min. The SAM treatment of the Au electrodes was performed following the steps described above. Solutions of C8‐BTBT (4 mg ml^−1^) and C16‐IDTBT (16 mg ml^−1^) were prepared in a 1:1 mixture of chlorobenzene and tetralin, and stirred at 80 °C for 2 h. The two solutions were then mixed in a 1:4 weight ratio of C8‐BTBT:C16‐IDTBT. The resulting blend solution was stirred overnight at room temperature. Before spin‐coating, the blended solution was stirred at 60 °C for 1 h. The blend solution was spun cast in two spin‐coating steps: i) 500 rpm for 10 s and ii) 2000 rpm for 30 s. After spin‐coating, the deposited blend films were thermally annealed at 120 °C for 3 min. The N3 solution was prepared in anhydrous chloroform (10 mg mL^−1^) and stirring overnight at 40 °C inside an N_2_‐filled glove box. Before spin‐coating, N3 solution was stirred at 50 °C for 1 h. After spin‐coating, the deposited N3 films were thermally annealed at 220 °C for 5 min. As the gate dielectric, Cytop (CTL‐809M) was spun‐cast at 4000 rpm for 90 s (with an acceleration of 400 rpm). The Cytop film was then thermally annealed at 50 °C for 2 h before the deposition of Al as the gate electrode. All the device fabrication steps were performed in an N_2_‐filled glove box.

### Characterization

The electrical characteristics of the OTFTs and inverters were monitored using a probe station placed in an N_2_‐filled glove box connected to an Agilent B1500A semiconductor parameter analyzer. The inverters were fabricated by connecting a p‐channel with an n‐channel transistor located on different glass substrates using external wires to form the equivalent inverter circuits shown in Figure 4f (inset). Photoelectron spectroscopy in air (PESA) was performed using a Riken Keiki PESA spectrometer (Model AC‐2). X‐ray photoelectron spectroscopy (XPS) studies were carried out in a Kratos Axis Supra DLD spectrometer equipped with a monochromatic Al Kα X‐ray source (hν = 1486.6 eV) operating at 150 W, a multi‐channel plate and delay line detector under a vacuum of ≈10^−9^ mbar. All the spectra were recorded using an aperture slot of 300 µm × 700 µm. Survey spectra were collected using a pass energy of 160 eV and a step size of 1 eV. A pass energy of 20 eV and a step size of 0.1 eV were used for the high‐resolution spectra. For XPS analysis, the samples were mounted in floating mode in order to avoid differential charging. Charge neutralization was required for all samples. Binding energies were referenced to the Au 4f at 84.0 eV. Scanning electron microscopy (SEM) measurements were carried out using a Nova NanoSEM 630 apparatus (FEI company) at an accelerating voltage of 10 kV, applied current of 56 pA, and magnification of ×100.000 and ×200.000.

### DFT Calculations

The results were obtained using the Density Functional Theory (DFT) Vienna Ab Initio Simulation Package (VASP) with a plane‐wave basis (and an energy cutoff of 500 eV), projector‐augmented waves (PAW), and the generalized gradient approximation (GGA) Perdew‐Burke‐Ernzerhof exchange‐correlation (xc) functional. To simulate the effect of molecular adsorption on Au, we used slabs with six Au layers in supercell geometry. The structures were rendered using VESTA software. Electrostatic potential averaging was performed using the VASPKIT.

## Conflict of Interest

The authors declare no conflict of interest.

## Supporting information



Supporting Information

## Data Availability

The data that support the findings of this study are available from the corresponding author upon reasonable request.
