# Supplementary material for: Thiol Carbazole Self‐Assembled Monolayers as Tunable Carrier Injecting Interlayers for Organic Transistors and Complementary Circuits
Source: Adv Mater. 2024 Dec 10;37(5):2413157. doi: 10.1002/adma.202413157 (PMC11795717; doi:10.1002/adma.202413157)
Supplement: Supplementary file 1 — Supporting Information [file ADMA-37-2413157-s001.docx]

Supporting Information

**Thiol Carbazole Self-Assembled Monolayers as Tunable Carrier Injecting Interlayers for Organic Transistors and Complementary Circuits**

*Mohamad Insan Nugraha,*^ƚ,^*^*^ Yu-Ying Yang,*^ƚ^ *Zhongzhe Liu, George T. Harrison, Ryanda Enggar Anugrah Ardhi, Yuliar Firdaus, Qiao He, Linqu Luo, Mohamed Nejib Hedhili, Marco Thaler, Zhaoheng Ling, Matthias Zeilerbauer, Laerte L. Patera, Leonidas Tsetseris, Shadi Fatayer,^*^Martin Heeney,^*^ Thomas D. Anthopoulos^*^*

Dr. M. I. Nugraha, Dr. Y. Yang, Dr. Z. Liu, Dr. G. T. Harrison, Dr. R. E. A. Ardhi, L. Luo, Z. Ling, Prof. S. Fatayer, Prof. M. Heeney, Prof. T. D. Anthopoulos

King Abdullah University of Science and Technology (KAUST), KAUST Solar Center (KSC), Thuwal 23955-6900, Saudi Arabia

Email: mohamad.nugraha@kaust.edu.sa; shadi.fatayer@kaust.edu.sa; martin.heeney@kaust.edu.sa; thomas.anthopoulos@kaust.edu.sa

Dr. M. I. Nugraha

Research Center for Nanotechnology Systems, National Research and Innovation Agency (BRIN), South Tangerang, Banten 15314, Indonesia

Dr. M. I. Nugraha

Collaboration Research Center for Advanced Energy Materials, National Research and Innovation Agency – Institut Teknologi Bandung, Jl Ganesha 10, Bandung, 40132, Indonesia

Dr. Y. Firdaus

Research Center for Electronics, National Research and Innovation Agency, Bandung 40135, Indonesia

Dr. Q. He

Department of Chemistry and Centre for Processable Electronics, Imperial College London, White City Campus, London W12 0BZ, United Kingdom (UK)

Dr. M. N. Hedhili

Core Labs, King Abdullah University of Science and Technology (KAUST), Thuwal 23955-6900, Saudi Arabia

M. Thaler, M. Zeilerbauer, Prof. L. L. Patera

Department of Physical Chemistry, University of Innsbruck, 6020 Innsbruck (Austria)

Prof. L. Tsetseris

Department of Physics, School of Applied Mathematical and Physical Sciences, National Technical University of Athens, 9 Heroon Polytechniou Street, Zografou Campus, Athens GR-15780, Greece

Prof. S. Fatayer

Applied Physics Program, Physical Science and Engineering Division, King Abdullah University of Science and Technology (KAUST), 23955-6900 Thuwal, Saudi Arabia

Prof. T. D. Anthopoulos

Henry Royce Institute and Photon Science Institute, Department of Electrical and Electronic Engineering, The University of Manchester, Oxford Road, Manchester, M13 9PL, United Kingdom

^ƚ^These authors contributed equally

**Materials Information:**

**2PACz**: [2-(9H-carbazol-9-yl)ethyl]phosphonic acid, **Br-2PACz**: (2-(3,6-dibromo-9H-carbazol-9-yl)ethyl)phosphonic acid, and **MeO-2PACz**: [2-(3,6-dimethoxy-9H-carbazol-9-yl)ethyl]phosphonic acid. **N3**: 2,2'-((2Z,2'Z)-((12,13-bis(3-ethylheptyl)-3,9-diundecyl-12,13-dihydro-[1,2,5]thiadiazolo[3,4-e]thieno[2'',3'':4',5']thieno[2',3':4,5]pyrrolo[3,2-g]thieno[2',3':4,5]thieno[3,2-b]indole-2,10-diyl)bis(methanylylidene))bis(5,6-difluoro-3-oxo-2,3-dihydro-1H-indene-2,1-diylidene))dimalononitrile. **C8-BTBT**: 2,7-dioctyl[1]benzothieno[3,2-b][1]benzothiophene. **C16-IDTBT**: polymer of 2,1,3-benzothiadiazole-4,7-diyl-co-4,4,9,9-tetrahexadecyl-4,9-dihydro-s-indaceno[1,2-b:5,6-b']dithiophene-2,7-diyl.

**Supplementary Methods**

All manipulations are performed under a nitrogen atmosphere with the use of standard Schlenk techniques unless otherwise stated. All the reagents were available commercially and used without further purification.

NMR spectra were performed in Bruker AVII 400 or 500 spectrometers in deuterated solvents at 298 K. Chemical shifts are referenced to the internal solvent resonance and reported as parts-per-million. Coupling constants (J) are expressed in Hertz (Hz) and multiplicities are indicated as singlet (s), doublet (d), triplet (t), quartet (q), or multiplet (m). High-resolution mass spectra (HRMS) were recorded on a Bruker COMPACT spectrometer. Matrix-assisted laser desorption/ionization time-of-flight mass spectrometry (MALDI-TOF MS) analysis was performed on a Bruker Autoflex III Smartbeam MALDI-TOF mass spectrometer. Chloroform was used as the solvent and trans-2-[3-(4-tert-butylphenyl)-2-methyl-2-propenylidene]malononitrile (DCTB) was used as the matrix.

**Sample preparation**


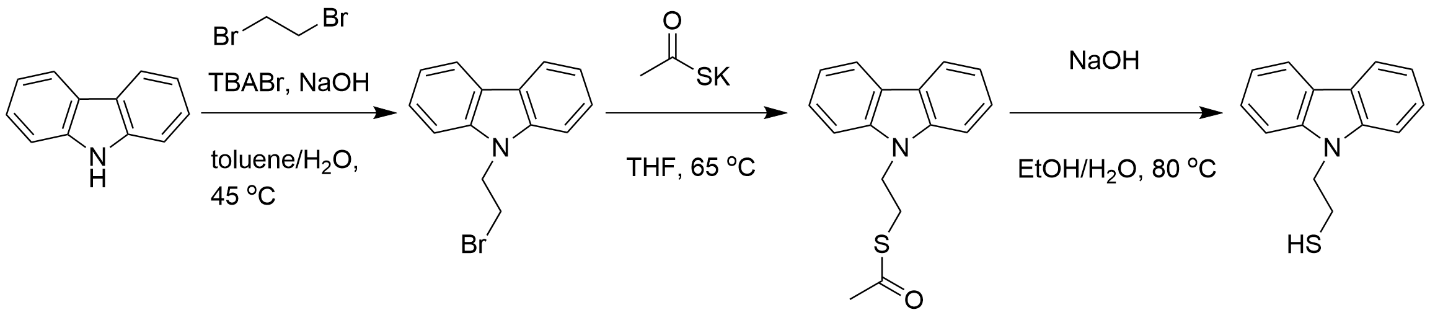


**9-(2-Bromoethyl)-9*H*-carbazole** was prepared according to the literature procedure.^1^ A mixture of 3,6-dibromocarbazole (1.00 g, 3.08 mmol), TBABr (0.020g, 0.06 mmol), 1,2-dibromoethane (5 mL, 57 mmol), 50% aqueous sodium hydroxide (8 mL) and toluene (10 mL) was heated at 75℃. After 8 h, the reaction mixture was cooled to room temperature, extracted with water and dichloromethane. The organic phase was dried over Na_2_SO_4_, then decompression evaporation and mixed with silica gel powder, and purified by silica gel chromatography column (Eluent: Petroleum ether/DCM, v/v=35:1~15:1) to obtain the product as a white solid in 67% (882 mg).^1^H NMR (400 MHz, CDCl_3_) δ = 8.12 (d, *J* = 8.0 Hz, 2H), 7.48-7.42 (m, 4H), 7.32-7.26 (m, 2H), 4.73 (t, *J* = 8.0 Hz, 2H), 3.70 (t, *J* = 8.0 Hz, 2H) ppm.

**S-[2-(9*H*-Carbazol-9-yl)ethyl] ethanethioate** A flask was charged with 9-(2-bromoethyl)-9*H*-carbazole (548 mg, 1.99 mmol), and was dried by heating at 60 °C under high vacuum for 4 h. After cooling to room temperature, it was transferred into the glovebox, and potassium thioacetate (682 mg, 6 mmol) was added. Dry THF (10 mL) was added and the resulting mixture was heated to reflux overnight. Upon cooling to room temperature, the solids were filtered off and washed with ethyl acetate. The filtrate was concentrated and purified by column chromatography over siliica (Eluent: DCM/ Petroleum ether = 1:1) to yield the desired product as a white solid in 82% yield (439 mg). ^1^H NMR (400 MHz, CDCl_3_) δ = 8.12 (d, *J* = 7.8 Hz, 2H), 7.60-7.44 (m, 4H), 7.32-7.21 (m, 2H), 4.49 (t, *J* = 8.0 Hz, 2H), 3.28 (t, *J* = 8.0 Hz, 2H), 2.42 (s, 3H) ppm.

**2-(9*H*-Carbazole-9-yl)ethane-1-thiol (2SCz)** A flask was charged with *S*-[2-(9*H*-carbazol-9-yl)ethyl]ethanethioate (1.00 g, 3.71 mmol, 1.00 eq.) and sodium hydroxide (445.5 mg, 11.14 mmol, 3.00 eq.). Ethanol (30 mL) and water (10 mL) were added and the mixture heated to 85 °C for 4 hours. After cooling to room temperature, the mixture was neutralized to pH = 7 by the addition of 1 M HCl (aq.), extracted with ethyl acetate (3 × 50 mL), dried over MgSO_4_, filtered and the solvent removed under reduced pressure. The crude product was recrystallized from MeOH as a yellowish-white solid in 65% yield (547.3 mg). ^1^H NMR (400 MHz, CDCl_3_) δ = 8.16 (d, *J* = 1.9 Hz, 2H), 7.59 (dd, *J* = 8.7, 1.9 Hz, 4H), 7.33 (d, *J* = 8.8 Hz, 2H), 4.48 (t, *J* = 7.0 Hz, 2H), 3.04-2.93 (m, 2H), 1.38 (t, *J* = 8.6 Hz, 1H) ppm. ^13^C NMR (101 MHz, CDCl_3_) δ = 140.20, 125.85, 123.03, 120.50, 119.33, 108.72, 46.37, 23.51. HRMS (ESI): *m/z* calcd for C_14_H_13_NS: 226.0690 [M-H]^+^ ; found: 224.0656 [M-H]^+^.


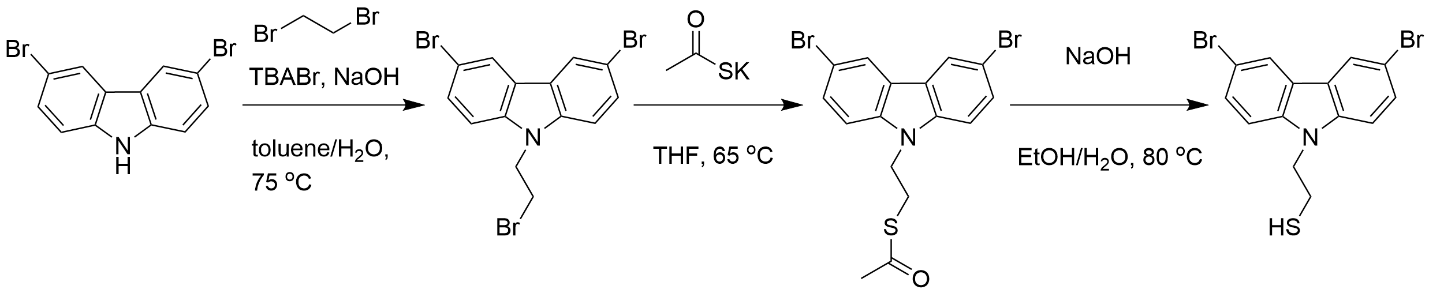


**3,6-Dibromo-9-(2-bromoethyl)-9*H*-carbazole** was prepared from 3,6-dibromo-9*H*-carbazole (1.30 g, 4.00 mmol) following a similar method to *S*-[2-(9*H*-carbazol-9-yl)ethyl]ethanethioate. The filtrate was concentrated and purified by column chromatography over siliica (Eluent: Petroleum ether/DCM, v/v=35:1~15:1) to afford the prouced as a while solid in 72% yield (1.24 g). ^1^H NMR (400 MHz, CDCl_3_) δ = 8.17 (d, *J* = 1.9 Hz, 2H), 7.60 (dd, *J* = 8.7, 1.9 Hz, 2H), 7.33 (d, *J* = 8.7 Hz, 2H), 4.68 (t, *J* = 7.2 Hz, 2H), 3.68 (t, *J* = 7.2 Hz, 2H) ppm.

***S*-[2-(3,6-Dibromo-9*H*-carbazol-9-yl)ethyl] ethanethioate** was prepared from 3,6-dibromo-9-(2-bromoethyl)-9*H*-carbazole (864 mg, 2.00 mmol) followed a similar method to *S*-[2-(9*H*-carbazol-9-yl)ethyl]ethanethioate. The filtrate was concentrated and purified by column chromatography over siliica (Eluent: DCM/ Petroleum ether = 1:1) to afford the prouced as a while solid in 79% yield (674 mg). ^1^H NMR (400 MHz, CDCl_3_) δ = 8.15 (d, *J* = 1.9 Hz, 2H), 7.60 (dd, *J* = 8.7, 1.9 Hz, 2H), 7.42 (d, *J* = 8.7 Hz, 2H), 4.41 (t, *J* = 8.0 Hz, 2H), 3.22 (t, *J* = 8.0 Hz, 2H), 2.41 (s, 3H) ppm.

**2-(3,6-Dibromo-9*H*-carbazole-9-yl)ethane-1-thiol (Br-2SCz)** was prepared from *S*-[2-(3,6-dibromo-9*H*-carbazol-9-yl)ethyl] ethanethioate (500 mg, 1.17 mmol) following a similar method to **2SCz** to afford the prouced as a while solid (234 mg, 52% yield). ^1^H NMR (400 MHz, CDCl_3_) δ = 8.16 (d, *J* = 1.9 Hz, 2H), 7.59 (dd, *J* = 8.7, 1.9 Hz, 2H), 7.34 (d, *J* = 8.7 Hz, 2H), 4.49 (t, *J* = 7.0 Hz, 2H), 3.02-2.93 (m, 2H), 1.38 (t, *J* = 8.6 Hz, 1H) ppm. ^13^C NMR (101 MHz, CDCl_3_) δ = 139.14, 129.27, 123.64, 123.42, 112.55, 110.49, 46.50, 23.48. HRMS (ESI): *m/z* calcd for C_14_H_11_NS: 383.8880 [M-H]^+^ ; found: 383.8926 [M-H]^+^.

**
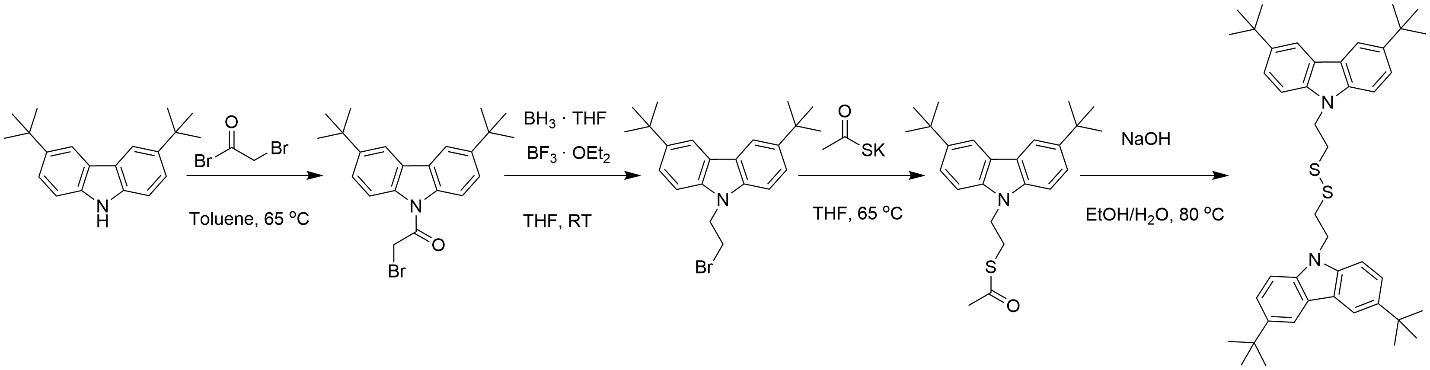
**

**2-Bromo-1-(3,6-di-*tert*-butyl-9*H*-carbazol-9-yl)ethan-1-one** A dry flask was charged with a mixture of 3,6-di-tert-butyl-9*H*-carbazole (1.83 g, 1.00 mmol) and dry toluene (50 mL). Bromoacetyl bromide (1.98 mL, 1.5 mmol) was added dropwise to the stirred mixture. The resulting suspension was heated at 65 ^o^C overnight. After cooling to room temperature, water (50 mL) was added slowly to quench excess bromoacetyl bromide. The resulting solution was extracted with ethyl acetate (30 mL x 2). The combined organics werewashed with a saturated aqueous solution of sodium bicarbonate (30 mL x 2) and brine (25 mL). The organic layer was dried over MgSO_4_ and filtered. The filtrate was concentrated in vacuo to afford the crude product. The crude product was recrystallized from MeOH to afford the product in 76% yield (2.62 g). ^1^H NMR (500 MHz, CDCl_3_) δ = 8.12 (d, *J* = 8.8 Hz, 2H), 8.01 (d, *J* = 2.0 Hz, 2H), 7.57 (dd, *J* = 8.8, 2.1 Hz, 2H), 4.61 (s, 2H), 1.48 (s, 18H) ppm. ^13^C NMR (126 MHz, CDCl_3_) δ = 165.37, 147.67, 136.44, 126.95, 125.15, 116.19, 116.05, 34.84, 31.69, 30.16.

**9-(2-Bromoethyl)-3,6-di-*tert*-butyl-9*H*-carbazole** To a stirred solution of 2-bromo-1-(3,6-di-tert-butyl-9*H*-carbazol-9-yl)ethan-1-one (3.15 g, 10.4 mmol) in BH_3_ · THF (1.0 M in THF, 20 mL, 20 mmol) was added BF_3_ · OEt_2_ (13.0 mL, 105 mmol) slowly at 0 ^o^C. Then, the reaction mixture was allowed to warm to room temperature. After stirring at room temperature for 24 h, the reaction solution was diluted with ethyl acetate (30 mL). The resulting solution was sequentially washed with a saturated aqueous solution of sodium bicarbonate (50 mL), water (50 mL), and brine (50 mL). The organic layer was dried over MgSO_4_ and filtered. After the filtrate was concentrated in vacuo, the crude product was recrystallized from MeOH to give a white solid in 62% yield (2.19 g). ^1^H NMR (400 MHz, CDCl_3_) δ = 8.13 (d, *J* = 2.0 Hz, 2H), 7.56 (dd, *J* = 8.6, 1.9 Hz, 2H), 7.36 (d, *J* = 8.6, 2H), 4.68 (t, *J* = 7.6, 2H), 3.68 (t, *J* = 7.6, 2H), 1.49 (s, 18H) ppm. ^13^C NMR (101 MHz, CDCl_3_) δ = 142.44, 138.46, 123.60, 123.05, 116.54, 107.83, 44.81, 34.73, 32.05, 28.35.

***S*-(2-(3,6-di-*tert*-butyl-9*H*-carbazol-9-yl)ethyl) ethanethioate** was prepared from 9-(2-bromoethyl)-3,6-di-*tert*-butyl-9*H*-carbazole (770 mg, 2.00 mmol) followed a similar method to *S*-[2-(9*H*-carbazol-9-yl)ethyl]ethanethioate. The filtrate was concentrated and purified by column chromatography over siliica (Eluent: DCM/ Petroleum ether = 1:1) to afford the prouced as a while solid in 76% yield (579 mg). ^1^H NMR (400 MHz, CDCl_3_) δ = 8.12 (d, *J* = 1.9 Hz, 2H), 7.56 (dd, *J* = 8.5, 1.9 Hz, 2H), 7.46 (d, *J* = 8.6 Hz, 2H), 4.43 (t, *J* = 7.6, 2H), 3.26 (t, *J* = 7.6, 2H), 2.43 (s, 3H), 1.49 (s, 18H).

**1,2-Bis(2-(3,6-di-*tert*-butyl-9*H*-carbazol-9-yl)ethyl)disulfane (tBu-2SCz)** was prepared from *S*-(2-(3,6-di-*tert*-butyl-9*H*-carbazol-9-yl)ethyl) ethanethioate (1 g, 2.63 mmol) following a similar method to **2SCz.** After neutralization, the mixture was filtered and washed (water: 15 mL x 3; methanol: 5 mL x 2) to afford the prouced as a yellowish white solid in 92% yield (821 mg). ^1^H NMR (400 MHz, CDCl_3_) δ = 8.12 (d, *J* = 1.6 Hz, 2H), 7.53 (dd, *J* = 8.6, 1.9 Hz, 2H), 7.36 (d, *J* = 8.6 Hz, 2H), 4.61 (t, *J* = 7.48, 2H), 3.10 (t, *J* = 7.48, 2H), 1.47 (s, 18H). ^13^C NMR (101 MHz, CDCl_3_) δ = 142.13, 138.55, 123.53, 122.95, 116.48, 107.78, 42.33, 35.84, 34.70, 32.05. MS (MALDI) ): *m/z* calcd for C_44_H_56_N_2_S_2_: 676.39 [M]^+^ ; found: 676.097 [M]^+^. HRMS (ESI): *m/z* calcd for C_22_H_28_NS: 338.1942 [M]^+^ ; found: 338.1977 [M]^+^. The molecular ion is observable in MALDI, but electrospray MS only shows the peak corresponding to the reduced species.

**Synthesis Discussion**

The synthesis of 2SCz and Br-2SCz used similar methodology in both cases, and proceeded smoothly. Attempts to apply the same methodology to tBu-2SCz were problematic however. Attempted reaction with excess 1,2-dibromoethane did afford the expected product, but a complex mixture in which some of reactants , as well as elimination products were present. Therefore the route was changed to initially reaction with bromoacetyl bromide, affording the mono-substituted product smoothly. A subsequent reduction afforded the product with an ethyl bromide group, which was converted to the thioacetate and then hydrolysed. The tBu-2SCz showed a much higher propensity to oxidise (to the disulfide) than the other two carbazole compounds, which were readily isolated as their thiols. tBu-2SCz was oxidised on work-up, and the thiol proton was clearly absent in the ^1^H NMR, and MALDI confirmed the presence of the disulfide dimer. The dimer was used directly in the formation of the SAM monolayer.


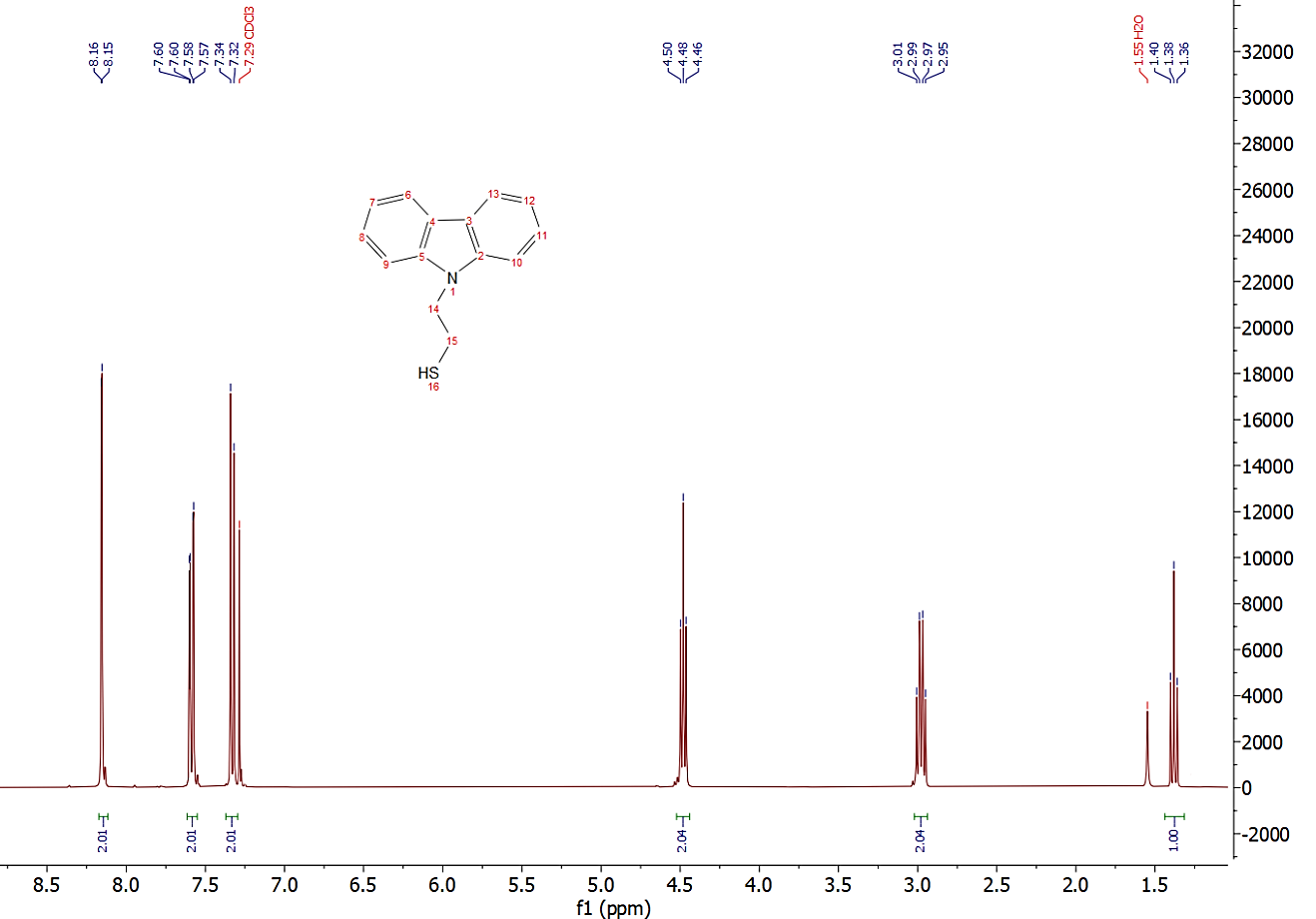


**Figure S1.** ^1^H NMR spectrum (400 MHz, CDCl_3_, 298 K) of **2SCz**.

**Figure S2.** ^13^C NMR spectrum (101 MHz, CDCl_3_, 298 K) of **2SCz**.


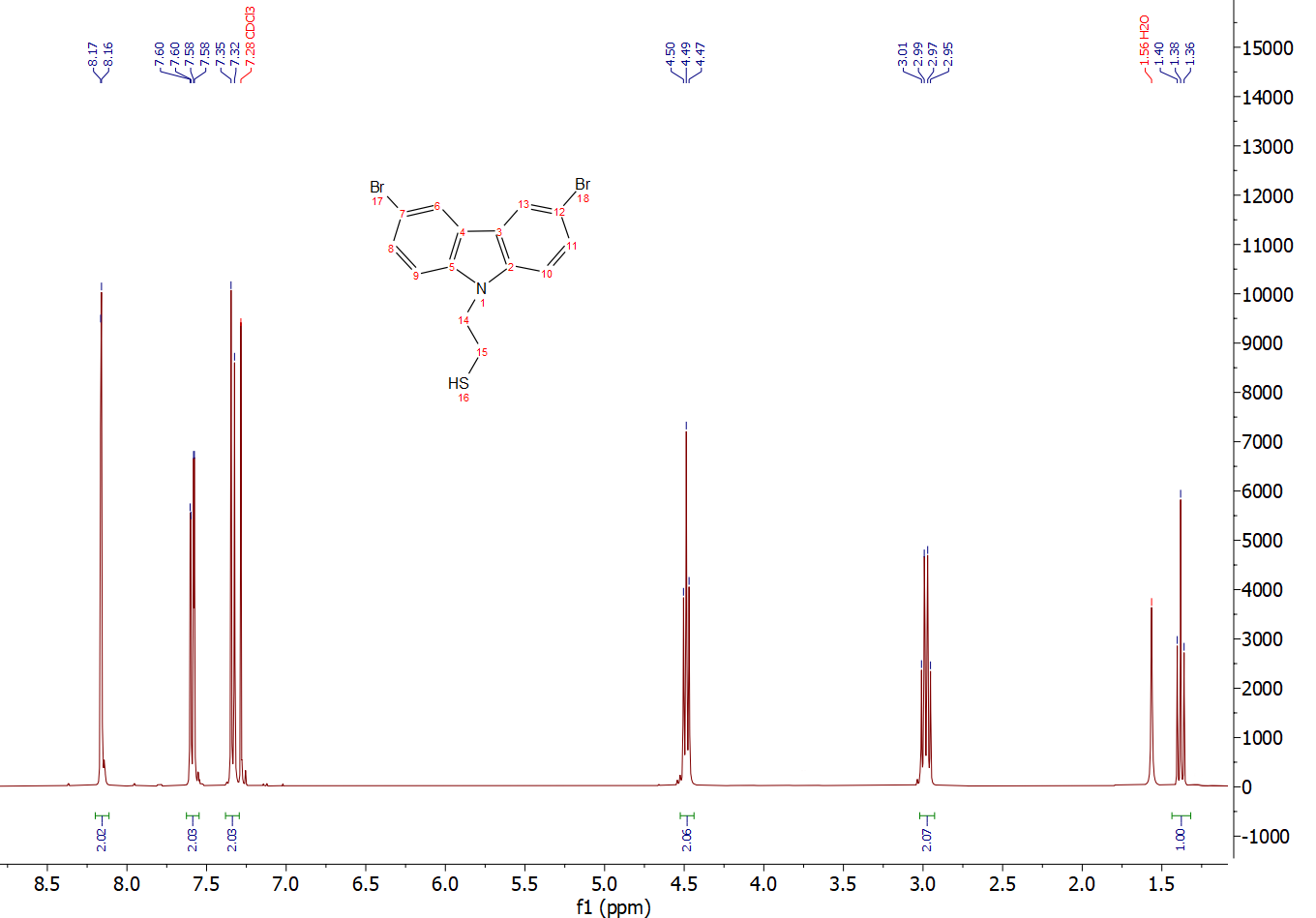


**Figure S3.** ^1^H NMR spectrum spectrum (400 MHz, CDCl_3_, 298 K) of **Br-2SCz**.

**Figure S4.** ^13^C NMR spectrum (101 MHz, CDCl_3_, 298 K) of **Br-2SCz**.


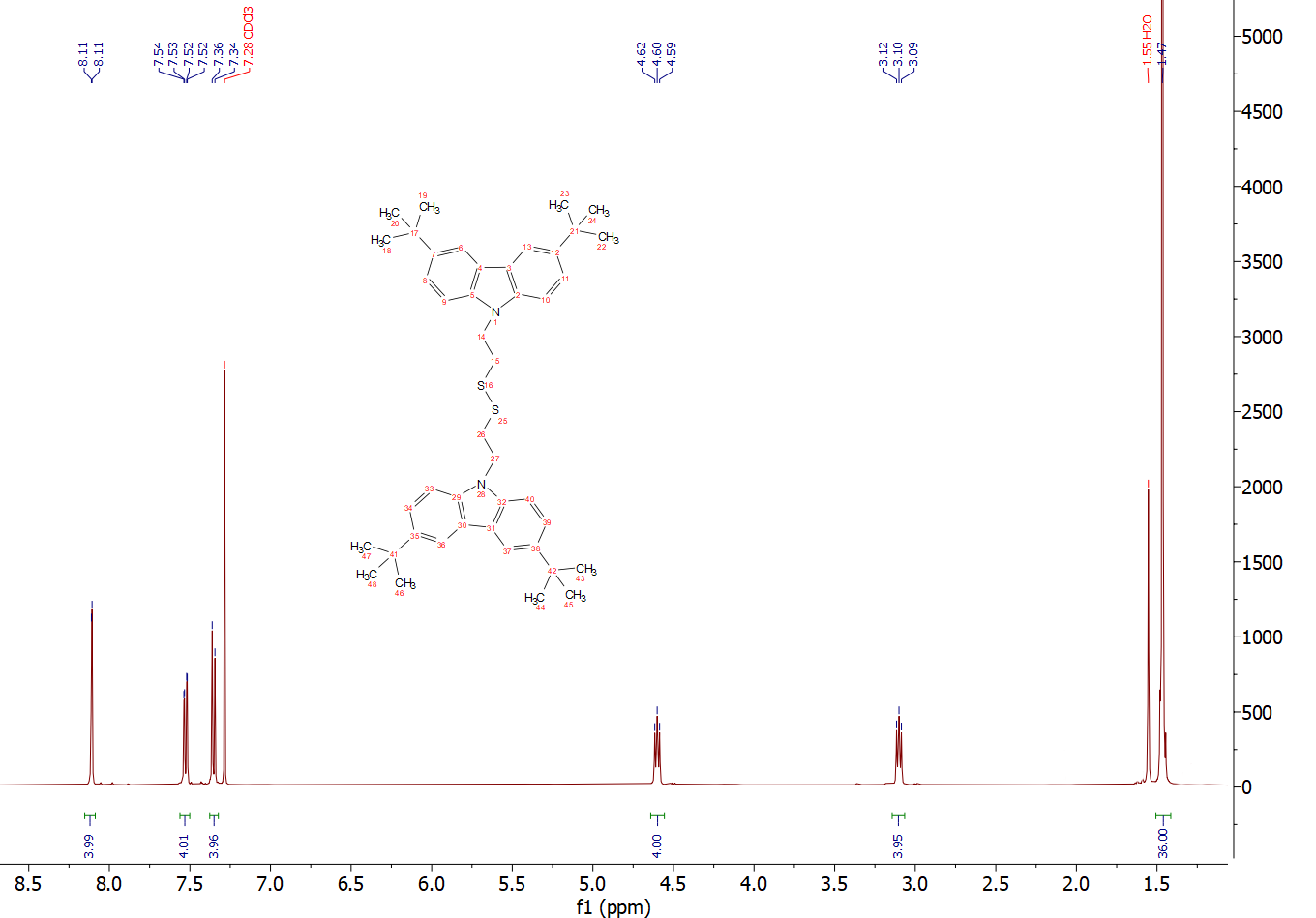


**Figure S5.** ^1^H NMR spectrum (400 MHz, CDCl_3_, 298 K) of **tBu-2SCz**.

**Figure S6.** ^13^C NMR spectrum (101 MHz, CDCl_3_, 298 K) of **tBu-2SCz**.

.
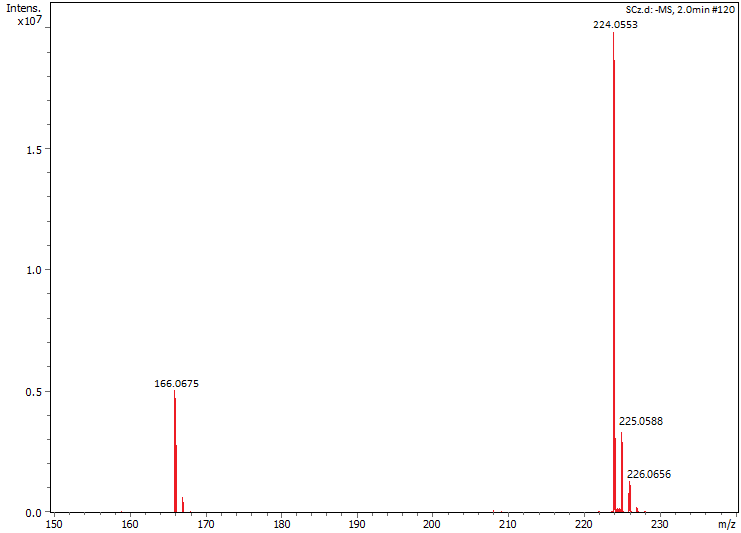


**Figure S7.** HRMS spectrum of **2SCz**.

**Figure S8.** HRMS spectrum of **Br-2SCz**.


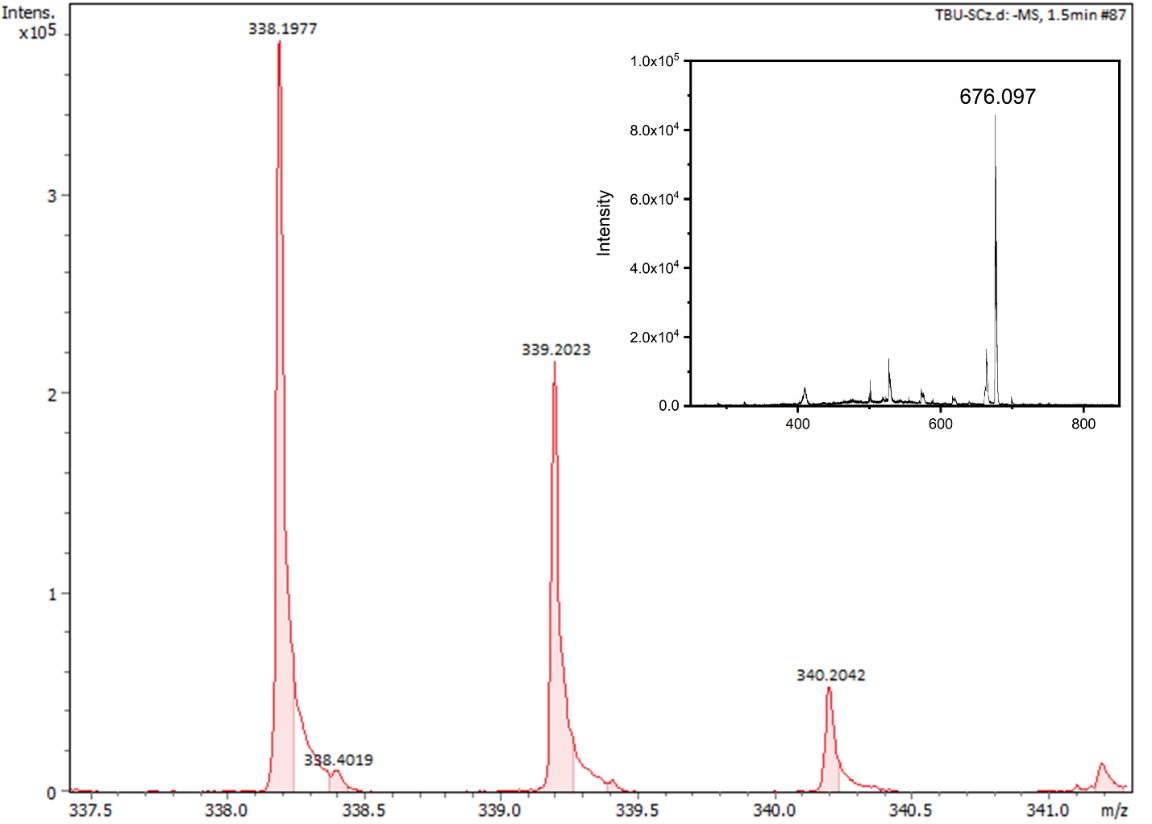


**Figure S9.** HRMS and MALDI-TOF MS (subfigure) spectra of **tBu-2SCz**.

**Figure S10.** Surface (plane-averaged) electrostatic potential (EP) of a Au(111) surface functionalized with different thiol SAMs as predicted by Density Functional Theory (DFT) calculation.

**Figure S11.** Detailed analysis of work functions of Au films functionalized with different thiol SAMs using PESA.

**Figure S12.** Surface potential of Au films functionalized with different thiol SAMs as revealed by Kelvin probe measurements (KPM).

**Table S1.** Work functions and dipole moments of Au functionalized with different thiol SAMs as calculated from DFT, PESA, and KPM.

| **Samples** | **Dipole moments per adsorbed molecule (Debye)** | **Work Function (eV)** | | |
| --- | --- | --- | --- | --- |
|  |  | **DFT** | **PESA** | **KPM** |
| PFBT | 0.36 (upward) | -5.33 | -5.34 | -5.33 |
| Br-2SCz | 0.39 (upward) | -5.45 | -5.48 | -5.47 |
| 2SCz | 0.93 (downward) | -4.87 | -4.85 | -4.84 |
| tBu-2SCz | 1.17 (downward) | -4.51 | -4.56 | -4.52 |

**Table S2.** Water contact angle and surface energy values of Au films treated with different SAMs.

| **SAMs** | **Contact Angle (^o^)** | **Surface Energy (mN/m)** |
| --- | --- | --- |
| Pristine | 66.13 (±0.04) | 43.13 ±0.17 |
| PFBT | 80.47 (±0.04) | 32.71 ±0.12 |
| Br-2SCz | 74.92 (±0.03) | 37.61 ±2.47 |
| 2SCz | 76.47 (±0.01) | 35.51 ±3.60 |
| tBu-2SCz | 91.14 (±0.95) | 29.61 ±1.72 |

**Figure S13.** Water contact angle of (a) pristine Au films and Au films functionalized with (b) PFBT, (c) Br-2SCz, (d) 2SCz, and (e) tBu-2SCz SAMs.

**Figure S14.** Optical constants [(a) extinction coefficient, k, and (b) refractive index, n] inferred from ellipsometry measurements for different SAMs studied in this work (PFBT, Br-2SCz, 2SCz, and tBu-2SCz). The SAMs were deposited on Au ((Si/SiO_2_ (48 nm)/Al(5 nm)/Au (79 nm)/SAMs)) and the ellipsometry data were fitted using the B-Spline model.

**Table S3.** Thickness of SAMs deposited on Au inferred from ellipsometry measurements (Si/SiO_2_ (48 nm)/Al(5 nm)/Au (79 nm)/SAMs). The ellipsometry data of the SAMs were fitted using B-Spline model. The Mean Squared Error (MSE) of the fitting is shown also in the table.

| **SAMs** | **Thickness from ellipsometry (nm)** | **MSE** | **Thickness from DFT (nm)** |
| --- | --- | --- | --- |
| PFBT | 0.66 | 2.5 | 0.735 |
| Br-2SCz | 2.23 | 2.1 | 1.026 |
| 2SCz | 2.06 | 2.3 | 0.949 |
| tBu-2SCz | 3.08 | 2.5 | - |

**Figure S15.** Scanning electron microscopy (SEM) of Au films functionalized with different thiol SAMs (magnification 100.000×).

**Figure S16.** Scanning electron microscopy (SEM) of Au films functionalized with different thiol SAMs (magnification 200.000×).

**Figure S17.** X-ray photoelectron spectroscopy (XPS) spectra of (a) F 1s core level in the PFBT-treated Au films, (b) Br 3d and (c) N 1s core levels in the Br-2SCz-treated Au films. The XPS spectra of the N 1s core level in (d) 2SCz and (e) tBu-2SCz-treated Au films.


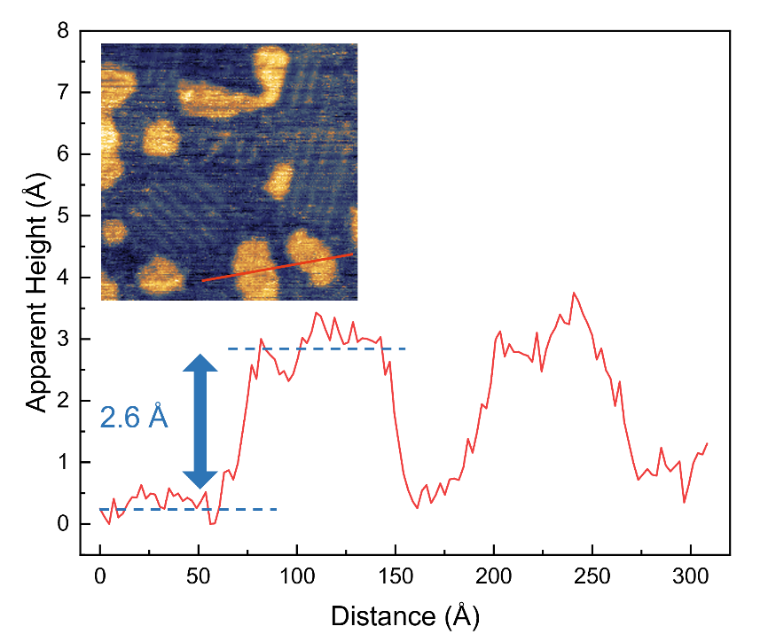


**Figure S18.** Line profile of PFBT/Au(111). Inset shows the STM image and the location of the line profile. Inset STM Image parameters: *V* = 250 mV, *I* = 50 pA.


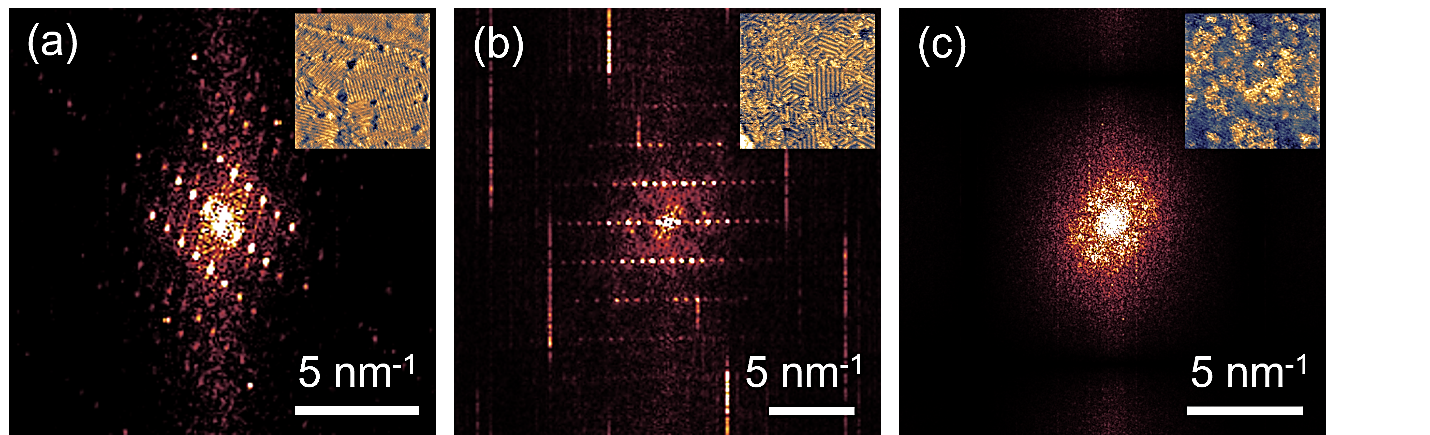


**Figure S19.** Fast Fourier transform (FFT) analysis of STM images of (a) 2SCz, (b) Br-2SCz, (c) tBu-2SCz; The superlattice of 2SCz and Br-2SCz was determined through FFT analysis of STM images and the lattice is superimposed on the STM images. 2SCz exhibits a c(2x10) adsorption superlattice on Au(111), whereas Br-2SCz exhibits a (2√7×√2)R30° adsorption superlattice on Au(111). The insets correspond to the respective STM image used to obtain the FFT image.

**Figure S20.** Detailed analysis of HOMO levels of (a) C8-BTBT, (b) C16-IDTBT, and (c) N3 organic semiconductors using PESA.

**Figure S21.** The transfer characteristics of C16-IDTBT/C8-BTBT blend OTFTs with pristine Au and PFBT-treated Au electrodes. The channel length and width of the devices are 30 µm and 1000 µm, respectively.

**Figure S22.** The transfer characteristics of the C16-IDTBT/C8-BTBT blend OTFTs employing Au S/D electrodes treated with different SAMs. The channel length and width of the devices are 30 µm and 1000 µm, respectively.

**Figure S23.** The I_D_^1/2^-V_G_ characteristics of the C16-IDTBT/C8-BTBT blend OTFTs employing Au S/D electrodes treated with different SAMs for the calculation of hole mobility. The channel length and width of the devices are 30 µm and 1000 µm, respectively.

**Figure S24.** The I_D_^1/2^-V_G_ characteristics of N3 OTFTs with (a) pristine Au and (b) tBu-2SCz-treated Au electrodes. The channel length and width of the devices are 30 µm and 1000 µm, respectively.

**Figure S25.** The Y-function characteristics of N3 OTFTs with (a) pristine Au and (b) tBu-2SCz-treated Au electrodes for the calculation of contact resistance. (c) The contact resistance of N3 OTFTs with pristine Au and tBu-2SCz-treated Au electrodes calculated using Y-function method.

**References**

**1** Lei, L. L. *et al.* Hierarchical Self-Assembly of Organic-Inorganic Hybrid Nanosheets to Construct Tubular Superstructures for Photocatalytic Degradation. *Acs Applied Nano Materials* **6**, 6270-6278 (2023).
